# Surgical Clinic

**Published:** 1855-01

**Authors:** 


### SURGICAL CLINIC.

Several interesting cases have been brought before the class of the present session to be operated upon.

The first was a gentleman from Henry County, in this State, who had suffered for some time from urinary calculi.

In May last he submitted to an operation, and although a stone was extracted, another still remained behind, which the patient soon perceived, and advised the physician who had operated of the fact ; who, as the patient himself says, intimated in a letter to him that it was impossible that it could be so.

He persisted in his opinion, and concluded to seek further counsel, and came for that purpose to the Hospital, and placed himself under the care of Prof. Hughes.

Owing to an increased curvature of the urethra in consequence of an incision into the rectum, and the subsequent cicatrization and union formed between the neck of the bladder and rectum, the former was drawn down, producing a serious difficulty in the introduction of the sound which required to be so modified in shape as to adapt it to the direction of the urethra. The sound once introduced, confirmed the opinion of the patient himself, of the existence of a calculus.

An operation was determined upon, and after some preparatory measures, he was brought upon the operating table for an operation. A few explanatory remarks were made to the class, and after the patient was brought under the influence of chloroform, Prof. Hughes proceeded with the operation, and from the time of the first incision, in four or five minutes the stone was extracted.—But little blood was lost during the operation. The bi-lateral operation was adopted in the case, and was selected because of the former injury to the rectum, and the consequent union there between it and the neck of the bladder.

The lithotome of Dupuytren was used in this operation, which is a great improvement over the gorget.

Prof. Hughes will report the case we understand in full, and if so, it shall appear in the pages of the next Journal. This operation, where no difficulties exist, is certainly a capital one; and he who enters upon it should feel himself competent to succeed, otherwise a prudent regard for the life of his fellow, and a respect for his own character, would restrain his hand. In this case there were embarrassing circumstances, and even with these, the operation was performed most deliberately, skillfully and promptly, and the result has placed Prof. Hughes as one among the very best operators, if not the very best in the West.

At this writing, ten days after the operation, the patient is doing well, and will return to his home during next week, if nothing unforeseen takes place to prevent.

